# Not Only High Number and Specific Comorbidities but Also Age Are Closely Related to Progression and Poor Prognosis in Patients With COVID-19

**DOI:** 10.3389/fmed.2021.736109

**Published:** 2022-01-07

**Authors:** Dafeng Liu, Yongli Zheng, Jun Kang, Dongmei Wang, Lang Bai, Yi Mao, Guifang Zha, Hong Tang, Renqing Zhang

**Affiliations:** ^1^Department of Internal Medicine, The Public and Health Clinic Centre of Chengdu, Chengdu, China; ^2^Center of Infectious Diseases, Sichuan University West China Hospital, Chengdu, China; ^3^The Public and Health Clinic Centre of Chengdu Substation, Chengdu New Emergent Infectious Disease Prevention and Control Workstation, Chengdu, China

**Keywords:** coronavirus disease 2019 (COVID-19), comorbidity, number, progression, prognosis

## Abstract

**Background:** Some patients with comorbidities and rapid disease progression have a poor prognosis.

**Aim:** We aimed to investigate the characteristics of comorbidities and their relationship with disease progression and outcomes of COVID-19 patients.

**Methods:** A total of 718 COVID-19 patients were divided into five clinical type groups and eight age-interval groups. The characteristics of comorbidities were compared between the different clinical type groups and between the different age-interval groups, and their relationships with disease progression and outcomes of COVID-19 patients were assessed.

**Results:** Approximately 91.23% (655/718) of COVID-19 patients were younger than 60 years old. Approximately 64.76% (465/718) had one or more comorbidities, and common comorbidities included non-alcoholic fatty liver disease (NAFLD), hyperlipidaemia, hypertension, diabetes mellitus (DM), chronic hepatitis B (CHB), hyperuricaemia, and gout. COVID-19 patients with comorbidities were older, especially those with chronic obstructive pulmonary disease (COPD) and cardiovascular disease (CVD). Hypertension, DM, COPD, chronic kidney disease (CKD) and CVD were mainly found in severe COVID-19 patients. According to spearman correlation analysis the number of comorbidities was correlated positively with disease severity, the number of comorbidities and NAFLD were correlated positively with virus negative conversion time, hypertension, CKD and CVD were primarily associated with those who died, and the above-mentioned correlation existed independently of age. Risk factors included age, the number of comorbidities and hyperlipidaemia for disease severity, the number of comorbidities, hyperlipidaemia, NAFLD and COPD for the virus negative conversion time, and the number of comorbidities and CKD for prognosis. Number of comorbidities and age played a predictive role in disease progression and outcomes.

**Conclusion:** Not only high number and specific comorbidities but also age are closely related to progression and poor prognosis in patients with COVID-19. These findings provide a reference for clinicians to focus on not only the number and specific comorbidities but also age in COVID-19 patients to predict disease progression and prognosis.

**Clinical Trial Registry:** Chinese Clinical Trial Register ChiCTR2000034563.

## Introduction

The worldwide pandemic caused by severe acute respiratory syndrome coronavirus 2 (SARS-CoV-2) infection, namely, coronavirus disease 2019 (COVID-19) presents a paramount and urgent threat to global health. (1–5) As of May 11, 2021, there were approximately 157,362,408 confirmed cases, including 3,277,834 deaths, reported worldwide (6). Although the overall prognosis of COVID-19 is good, (1–5) some patients with comorbidities or rapid disease progression have a poor outcome (7–12).

Previous studies have shown that approximately 66.67~70.70% of COVID-19 patients have comorbidities; common comorbidities are hypertension, cardiovascular disease (CVD), diabetes mellitus (DM), chronic obstructive pulmonary disease (COPD), malignancy, chronic kidney disease (CKD), and obesity (13–15). DM, hypertension, CVD, active malignancy, and a high number of comorbidities are risk factors for a worse outcome (16–19). DM and hypertension, or CVD are common underlying diseases related to death in hospitalized cases (14).

The distribution characteristics of comorbidities in different age intervals and clinical types and whether other comorbidities are also related with the progression and prognosis of COVID-19 are unknown and worth studying.

## Methods

### Subjects

This study had a cross-sectional research design.

**Figure 1:**
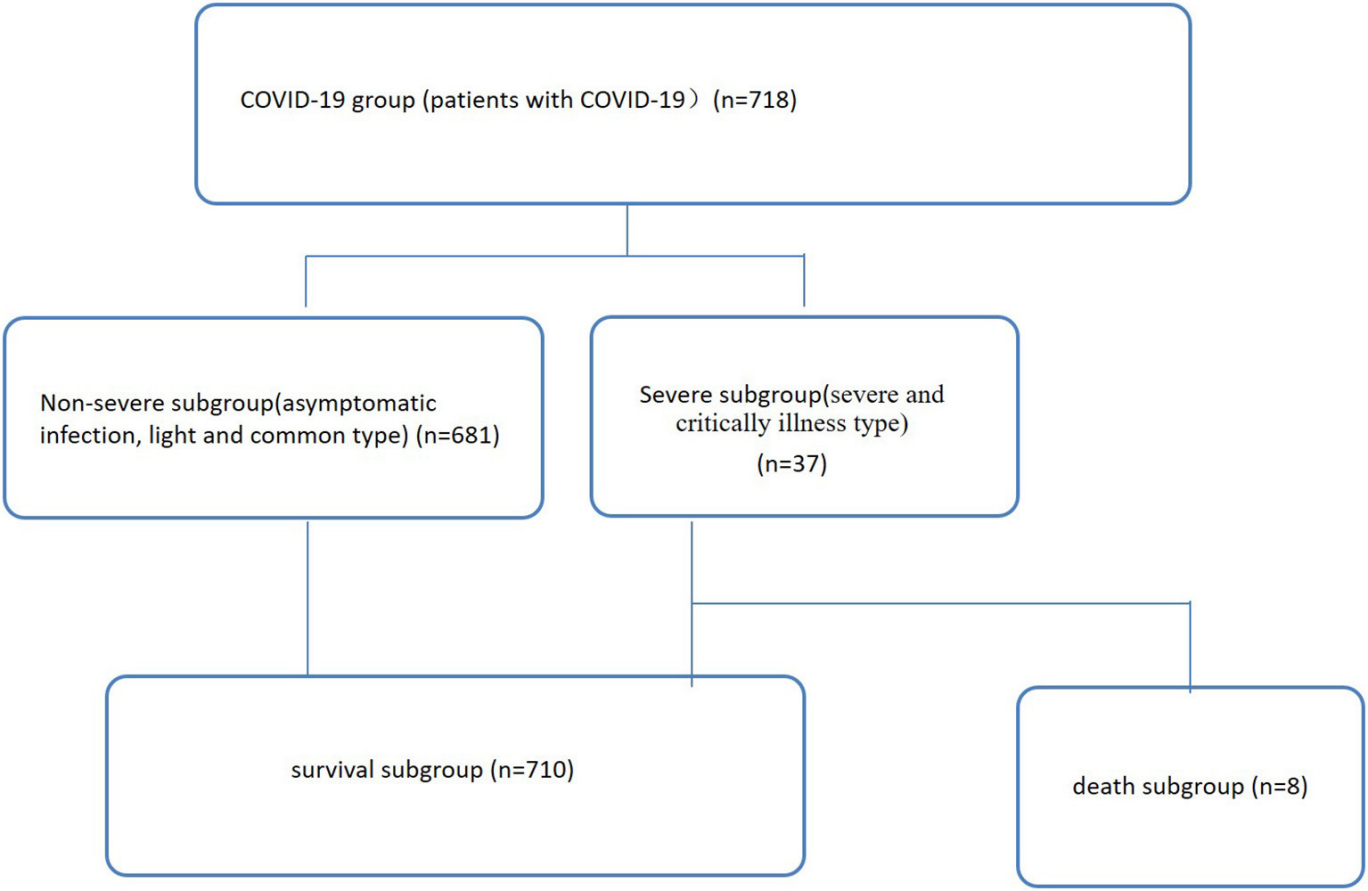
Patient data (*n* = 718). Non-severe refers to the clinical type of COVID-19 that is asymptomatic, light and common. Severe refers to the clinical type of COVID-19 that is associated with severe and critical illness.

In total, 718 COVID-19 patients from the hospital isolation ward who presented to the Public and Health Clinic Center of Chengdu from January 16, 2020, to April 30, 2021, were retrospectively recruited (Figure 1). The Ethics Committee of the Public and Health Clinic Center of Chengdu approved this study (ethics approval number: PJ-K2020-26-01). Written informed consent was waived by the Ethics Commission of the designated hospital because this study was related to emerging infectious diseases.

### Clinical Typing, Disease Diagnosis and Cure Criteria

The criteria for COVID-19 clinical typing and disease diagnosis were based on the seventh Trial Version of the Novel Coronavirus Pneumonia Diagnosis and Treatment Guidance (7).

### Grouping Standards

Seven hundred eighteen COVID-19 patients were enrolled (Figure 1), including 681 and 37 non-severe (asymptomatic infection, light and common) and severe (severe and critical illness) cases, respectively (Table 1, Figure 1). Of these patients, 710 and eight cases were divided into a survival subgroup (those who survived) and a death subgroup (those who died), respectively (Table 1, Figure 1).

**Table 1:** Baseline information (*n* = 718).

**Variables**	**χ ± SD or case(%)**	**Range**
Age (years)	38.48 ± 14.15	0.17~87
Male (case, %)	529(73.68)	
Duration (day)	1.74 ± 1.20	1~30 day
Virus negative conversion time (day)	15.48 ± 11.18	2~53 day
In-hospital time (day)	18.28 ± 11.16	2~56 day
**Disease severity**		
Non-severe (case, %)	681(94.85)	
Severe (case, %)	37 (5.15)	
**Prognosis**		
Survival (case, %)	710(98.89)	
Death (case, %)	8(1.11)	

**Figure 2:**
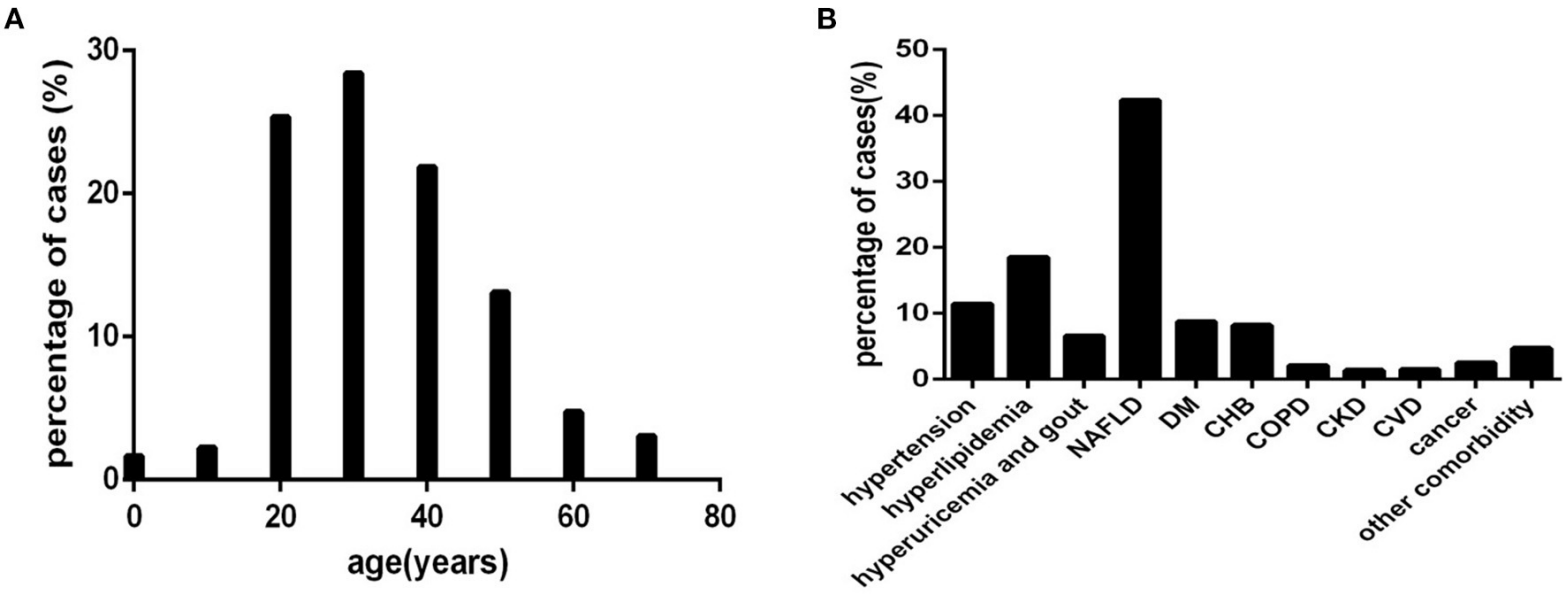
The distribution characteristics of age and comorbidities in COVID-19 patients (*n* = 718). COVID-19, coronavirus disease 2019. **(A)** age. **(B)** comorbidity.

Based on every 10 years as an age interval, 12, 16, 182, 204, 157, 94, 34, and 29 cases were divided into eight age-interval subgroups of 0~10, 10~20, 20~30, 30~40, 40~50, 50~60, 60~70, and >70 years, respectively (Figure 2A).

Based on the type of comorbidity, 82, 133, 47, 304, 63, 59, 15, 10, 11, 18, and 34 cases were divided into a hypertension subgroup (those with hypertension), hyperlipidaemia subgroup (those with hyperlipidaemia), hyperuricaemia and gout subgroup (those with hyperuricaemia and gout), non-alcoholic fatty liver disease (NAFLD) subgroup (those with NAFLD), DM subgroup (those with DM), chronic hepatitis B (CHB) subgroup (those with CHB), COPD subgroup (those with COPD), CKD subgroup (those with CKD), CVD subgroup (those with CVD), cancer abovementioned subgroup (those with cancer), and other comorbidity subgroup (those with abovementioned comorbidity) (Figure 2B), respectively.

The 718 COVID-19 patients were also divided according to the number of comorbidities into none comorbidity subgroup (patients without comorbidities), one comorbidity subgroup (patients with one comorbidity), two comorbidity subgroup (patients with two comorbidities), and three and more comorbidity subgroup (patients with three and more comorbidities), with 253,193,127 and 145 cases, respectively (Figure 4A).

According to clinical type, 234, 73, 371, 18 and 19 cases were divided into an asymptomatic infection group (patients belonging to symptom infection clinical type), a light group (patients belonging to light clinical type), a common group (patients belonging to common clinical type), a severe group (patients belonging to severe clinical type), and a critically ill group (patients belonging to critically illness clinical type), respectively (Figure 4B).

### Data Collection

Demographic data, clinical data, and lymphocyte and subset counts for all 718 cases were collected and used to establish databases. The authenticity, accuracy and completeness of the data were strictly controlled.

### Statistical Analysis

Statistical analyses were performed using GraphPad Prism 8 (GraphPad, CA, USA) and SPSS 26.0 (SPSS, Chicago, IL, USA). Measurement data are expressed as x ± SD, and ANOVA was used for multigroup comparisons of the homogeneity of variance and normally distributed data. A least significant difference (LSD) *t*-test was used for further comparisons between two groups. An independent-sample *t*-test was employed for comparisons between two groups. A percentage or proportion was used to express enumeration data, and a chi-square test and Fisher's exact test were applied for comparisons of these data. Spearman correlation analysis and partial correlation analysis were used for two-factor correlation analysis. Receiver operating characteristic (ROC) analysis for age was performed to assess the ability to distinguish between non-severe and severe patients and between surviving patients and those who died. Statistical significance was defined as *P* < 0.05.

## Results

### General Conditions

Approximately 5.16% (37/718) (Table 1, Figure 5A) of patients had severe COVID-19, and 1.11% (8/718) (Table 1, Figure 5B) of severe COVID-19 patients died.

For the distribution characteristics of age, approximately 91.23% (655/718) (Figure 2A) of COVID-19 patients were younger than 60 years old. A small number of patients were younger than 20 years old or older than 60 years old (Figure 2A).

**Figure 3:**
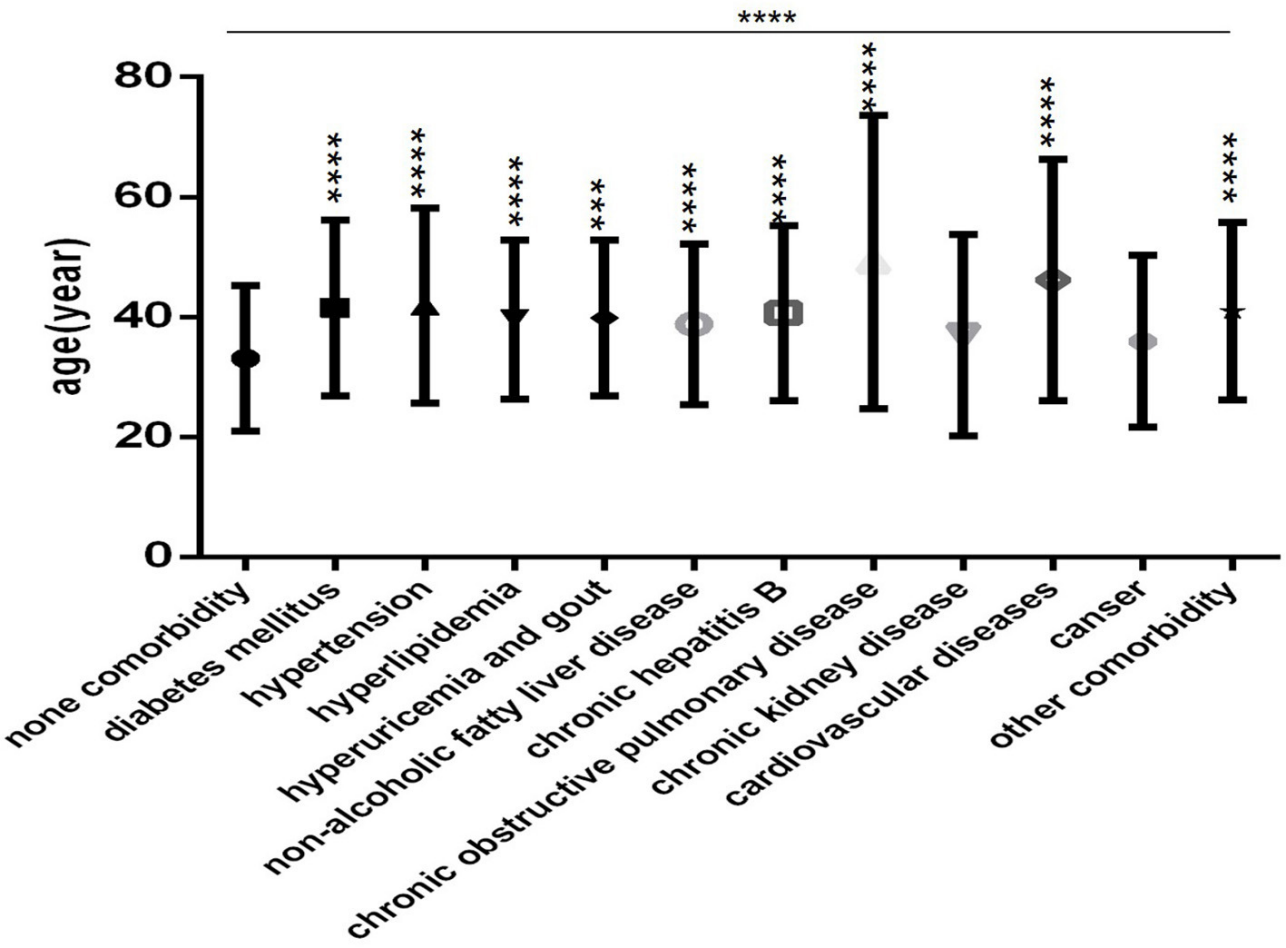
Comparison of age among the no comorbidity group and each comorbidity group (*n* = 718; 0, 1, 2, 3 or more comorbidities groups, *n* = 253, 193, 127, and 145, respectively). COVID-19, coronavirus disease 2019. Unpaired one-way ANOVA was used for intergroup comparisons (*P* < 0.0001). Unpaired *t*-tests were used for comparisons with the control group, ***P < 0.001, ****P < 0.0001.

Patients in each comorbidity subgroup were older than those in the none comorbidity subgroup (Figure 3), especially those with COPD and CVD (Figure 3). Except for the CKD subgroup and the cancer subgroup, the differences were statistically significant (all *P* < 0.001).

**Figure 4:**
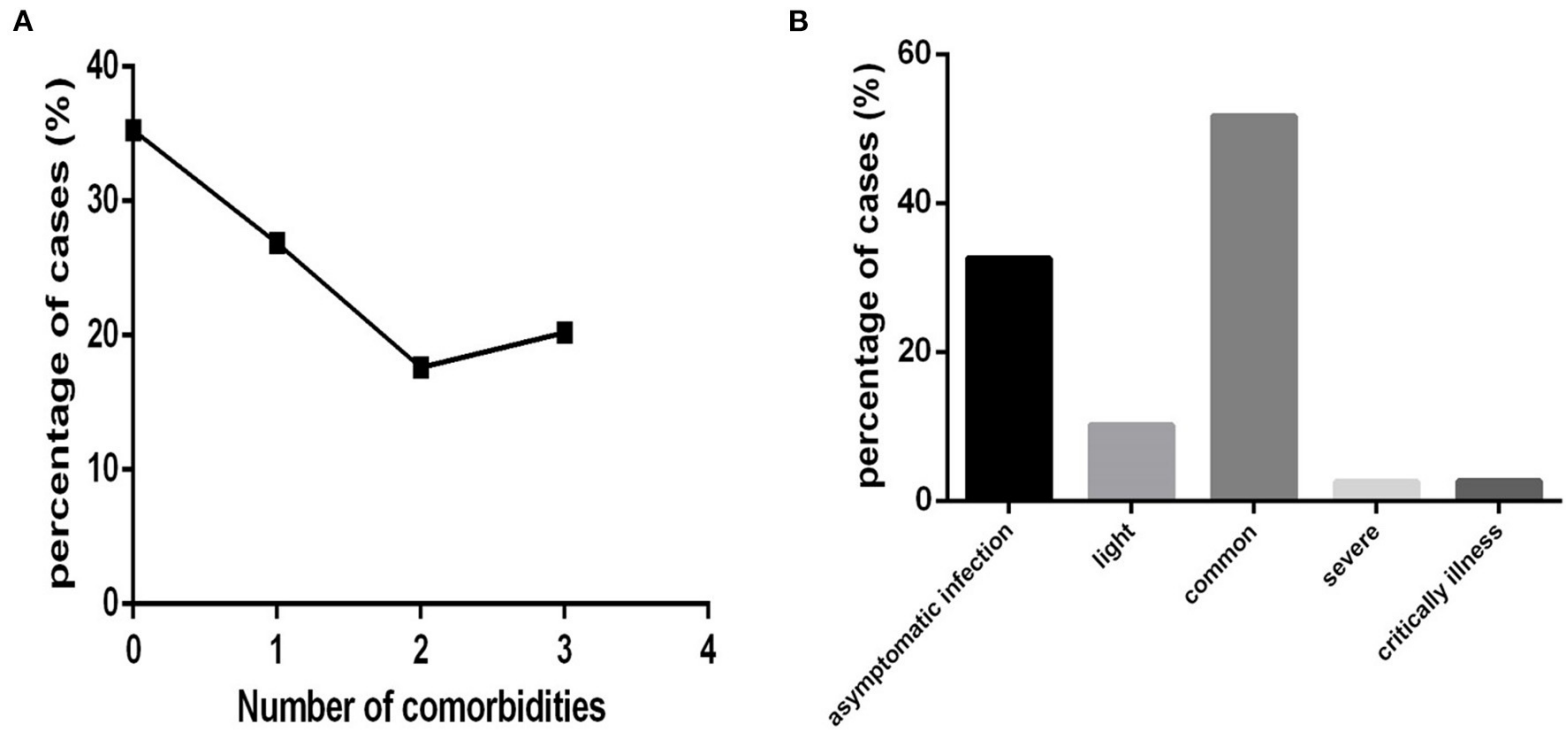
The distribution characteristics of the number of comorbidities and clinical type among COVID-19 patients (*n* = 718). COVID-19, coronavirus disease 2019. **(A)** Number of comorbidities. **(B)** Clinical type.

In this COVID-19 cohort, the order of clinical type according to the number of cases was as follows: common, asymptomatic infection, light, critical illness and severe. The percentages were 51.67, 32.59, 10.17, 2.65, and 2.51%, respectively (Figure 4B).

**Figure 5:**
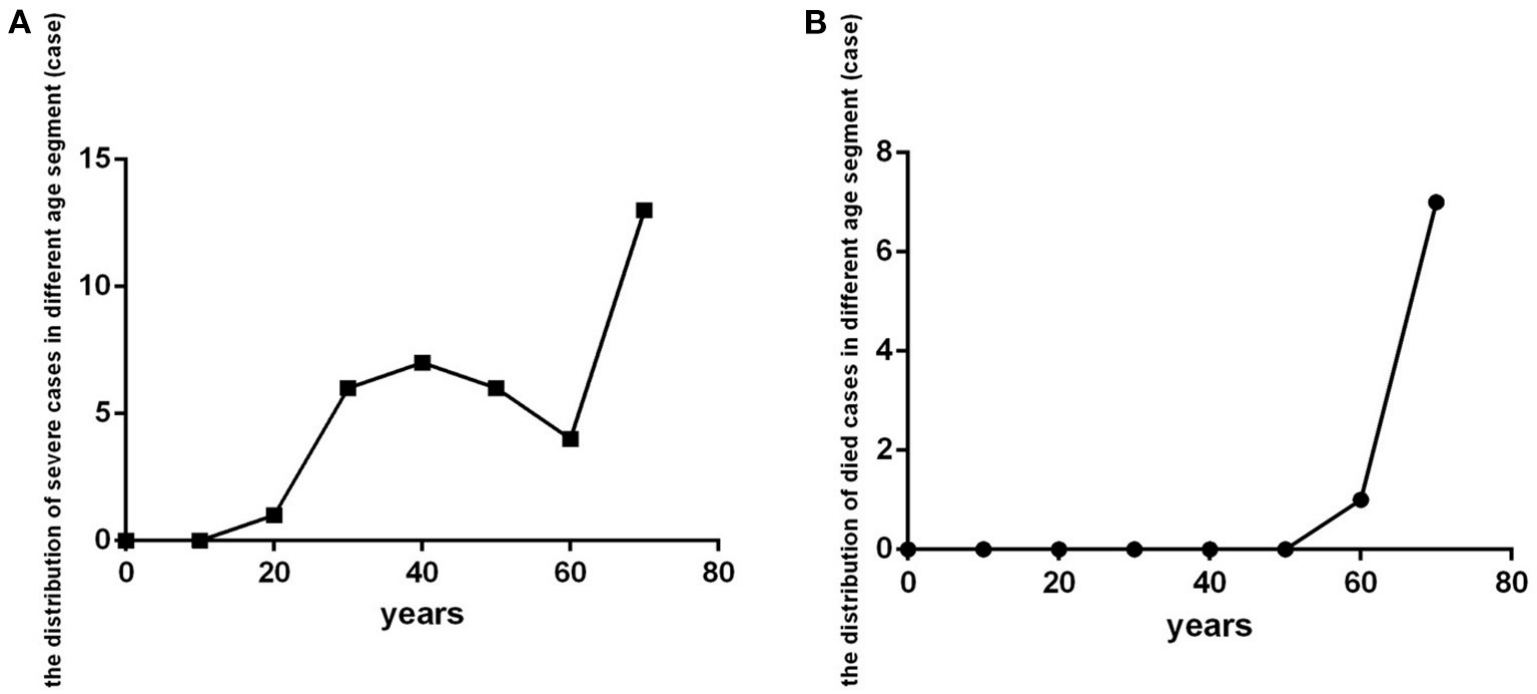
The distribution of patients with severe COVID-19 and who died in different age-interval groups (*n* = 718). COVID-19, coronavirus disease 2019. **(A)** Severe cases. **(B)** dead cases.

Severe cases (critical illness and severe clinical type) were distributed in age-interval subgroups older than 20 years, especially in the subgroup of patients older than 70 years (Figure 5A). Those who died were in the older than 60 age-interval subgroups, especially in those older than 70 (Figure 5B).

### The Distribution Characteristics of Comorbidities in Different Clinical Type Groups and Different Age-Interval Subgroups

Approximately 64.76% (465/718) (Figure 4A) of COVID-19 patients had one or more comorbidities (except for cases in none comorbidity group), and 37.88% (272/718) of patients had two or more comorbidities (except for cases in none comorbidity group and in one comorbidity group).

Common comorbidities were NAFLD, hyperlipidaemia, hypertension, DM, CHB, hyperuricaemia, and gout (Figure 2B). Cancer, COPD, CVD, CKD, and other comorbidities were rare (Figure 2B).

**Figure 6:**
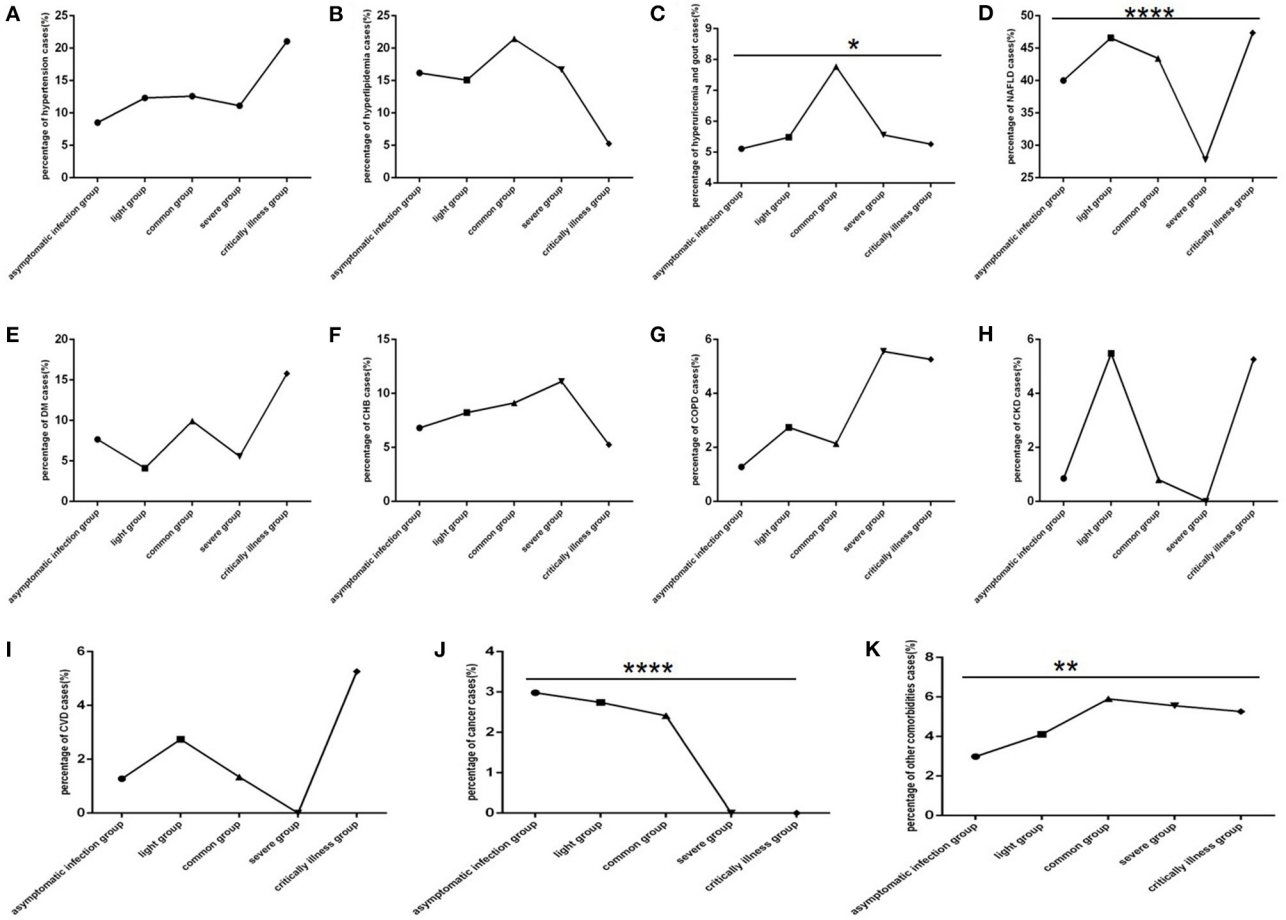
Comparison of the percentage of each comorbidity case among different clinical type groups (*n* = 718; asymptomatic infection, light, common, severe and critically ill groups, *n* = 234, 73, 371, 18, and 19, respectively). COVID-19, coronavirus disease 2019; NAFLD, non-alcoholic fatty liver disease; DM, diabetes mellitus; CHB, chronic hepatitis B; COPD, chronic obstructive pulmonary disease; CKD, chronic kidney disease; CVD, cardiovascular disease. **(A)** Hypertension. **(B)** Hyperlipidaemia. **(C)** Hyperuricaemia and gout. **(D)** NAFLD. **(E)** DM. **(F)** CHB. **(G)** COPD. **(H)** CKD. **(I)** CVD. **(J)** Cancer. **(K)** Other. Unpaired one-way ANOVA was used for intergroup comparisons (D, J, *P* all < 0.0001; K, *P* all < 0.01; C, *P* < 0.05; A, B, E, F, G, H, I, all *P* >0.05). *p < 0.05, **p < 0.01, ****p < 0.0001.

Among COVID-19 patients, hypertension, DM, COPD and CVD were mainly found in patients with the critically ill clinical type (Figures 6A,E,G,I), CKD and CVD were mainly found in patients with the common and severe clinical type (Figures 6H,I), hyperuricaemia and gout (Figure 6C) were mainly found in patients with the common clinical type, hyperlipidaemia, CHB, and other comorbidities were found in all clinical type (Figures 6B,F,K), cancer was mainly found in non-severe patients (Figure 6J). NAFLD (Figure 6D) was rare in those with the severe clinical type.

**Figure 7:**
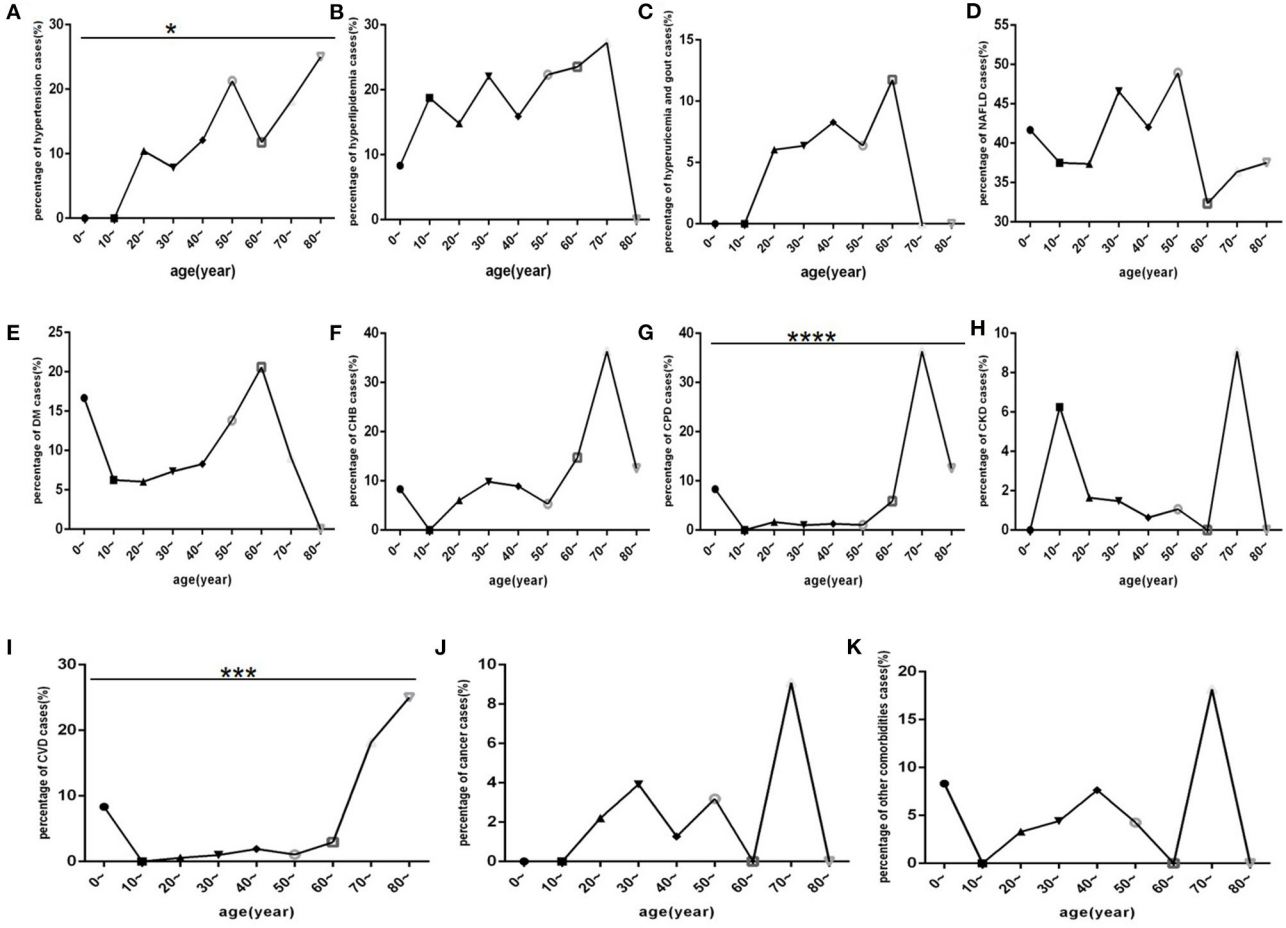
Comparison of the percentage of each comorbidity case among different age-interval groups (*n* = 718). COVID-19, coronavirus disease 2019; NAFLD, non-alcoholic fatty liver disease; DM, diabetes mellitus; CHB, chronic hepatitis B; COPD, chronic obstructive pulmonary disease; CKD, chronic kidney disease; CVD, cardiovascular disease. **(A)** Hypertension. **(B)** Hyperlipidaemia. **(C)** Hyperuricaemia and gout. **(D)** NAFLD. **(E)** DM. **(F)** CHB. **(G)** COPD. **(H)** CKD. **(I)** CVD. **(J)** Cancer. **(K)** Other. Unpaired one-way ANOVA was used for intergroup comparisons (G, *P* < 0.0001; I, *P* < 0.001; A, *P* < 0.05; B, C, D, E, F, H, J, K, all *P* > 0.05). *p < 0.05, ***p < 0.001, ****p < 0.001.

Among COVID-19 patients, hypertension, CHB, COPD and CVD (Figures 7A,F,G,I) were mostly distributed in those aged 50 and 80 years old. Cancer, CKD and other comorbidities (Figures 7H,J,K) mostly occurred in patients older than 70 years. DM, hyperlipidaemia, hyperuricaemia and gout, and NAFLD (Figures 7B–E) were mostly distributed in patients aged 20 and 70 years.

### The Relationship of Comorbidities With Disease Progression and Prognosis in COVID-19 Patients

In the severe group, patients were older than those in the non-severe group and had a greater number of comorbidities (Table 2) (all *P* < 0.0001). However, there were no differences in comorbidities between the two groups (Table 2) (*P* > 0.05).

**Table 2:** Comparison of age and comorbidities between the non-severe group and the severe group (*n* = 718).

**Variable**	**Non-severe group (*n* = 681)**	**Severe group (*n* = 37)**	***t*** Score or χ*^**2**^* **score**	***P*** **score**
Age (year)	37.44 ± 13.14	57.70 ± 18.08	*t* =-8.940	<0.001
Number of comorbidities	1.19 ± 1.12	2.53 ± 0.94	*t* =-7.027	<0.001
Hypertension (case, %)	76 (11.16)	6 (16.22)	χ*^2^* =-0.141	0.347
Hyperlipidaemia (case, %)	129 (18.94)	4 (10.81)	χ*^2^* =-1.239	0.215
Hyperuricaemia and gout (case, %)	45 (6.61)	2 (5.41)	χ*^2^* =-0.288	0.773
Non-alcoholic fatty liver disease (case, %)	290 (42.58)	14 (37.84)	χ*^2^* =-0.569	0.570
Diabetes mellitus (case, %)	59 (8.66)	4 (10.81)	χ*^2^* =-0.449	0.653
Chronic hepatitis B (case, %)	56 (8.22)	3 (8.11)	χ*^2^* =-0.025	0.980
Chronic obstructive pulmonary disease (case, %)	13 (1.91)	2 (5.41)	χ*^2^* =-1.447	0.148
Chronic kidney disease (case, %)	9 (1.32)	1 (2.70)	χ*^2^* =-0.698	0.485
Cardiovascular diseases (case, %)	12 (1.76)	2 (5.41)	χ*^2^* =-1.560	0.119
Cancer (case, %)	18 (2.62)	0 (0.00)	χ*^2^* =-1.001	0.317
Other (case, %)	49 (7.20)	3 (8.11)	χ*^2^* =-0.208	0.835

In the dead group, patients were older than those in the surviving group and had a greater number of comorbidities, more hypertension, more chronic kidney disease and more cardiovascular diseases (Table 3) (all *P* < 0.05). No differences in other comorbidities between the two groups were detected (Table 3) (*P* > 0.05).

**Table 3:** Comparison of age and comorbidities between the survival group and the non-surviving group (*n* = 718).

**Variable**	**Survival group (*n* = 710)**	**Dead group (*n* = 8)**	***t*** **Score or χ*^**2**^* score**	***P*** **score**
Age (year)	38.10 ± 13.67	77.14 ± 6.69	*t* =-7.543	<0.001
Number of comorbidities	1.24 ± 1.13	3.00 ± 0.00	*t* =-4.075	<0.001
Hypertension (case, %)	79 (11.13)	3 (37.50)	χ*^2^* =-2.626	0.009
Hyperlipidaemia (case, %)	133 (18.73)	0 (0.00)	χ*^2^* =-1.267	0.205
Hyperuricaemia and gout (case, %)	47 (6.62)	0 (0.00)	χ*^2^* =-0.703	0.482
Non-alcoholic fatty liver disease (case, %)	301 (42.39)	3 (37.50)	χ*^2^* =-0.028	0.978
Diabetes mellitus (case, %)	63 (8.87)	0 (0.00)	χ*^2^* =-0.824	0.410
Chronic hepatitis B (case, %)	58 (8.17)	1 (12.50)	χ*^2^* =-0.587	0.557
Chronic obstructive pulmonary disease (case, %)	15 (2.11)	0 (0.00)	χ*^2^* =-0.388	0.698
Chronic kidney disease (case, %)	9 (1.27)	1 (12.50)	χ*^2^* =-2.923	0.003
Cardiovascular diseases (case, %)	13 (1.83)	1 (12.50)	χ*^2^* =-2.370	0.018
Cancer (case, %)	18 (2.54)	0 (0.00)	χ*^2^* =-0.426	0.670
Other (case, %)	52 (7.32)	0 (0.00)	χ*^2^* =-0.742	0.408

In ≥60 years group, patients had a greater number of comorbidities, more CHB, more COPD and more CVD (Table 4) (all *P* < 0.05). No differences in other comorbidities between <60 years group and ≥60 years group were detected (Table 4) (*P* > 0.05).

**Table 4:** Comparison of comorbidities between <60 years group and ≥60 years group (*n* = 718).

**Variable**	** <60 years group (*n* = 667)**	**≥60 years group (*n* = 51)**	***t*** **score or χ*^**2**^* score**	***P*** **score**
Number of comorbidities	1.17 ± 1.11	2.45 ± 0.97	*t* =-8.022	<0.001
Hypertension (case, %)	74 (10.93)	8 (15.69)	χ*^2^* =-0.993	0.321
Hyperlipidaemia (case, %)	122 (18.02)	11 (21.57)	χ*^2^* =-0.580	0.562
Hyperuricaemia and gout (case, %)	43 (6.35)	4 (7.84)	χ*^2^* =-0.388	0.698
Non-alcoholic fatty liver disease (case, %)	287 (42.39)	17 (33.33)	χ*^2^* =-1.350	0.177
Diabetes mellitus (case, %)	55 (8.12)	8 (15.69)	χ*^2^* =-1.809	0.070
Chronic hepatitis B (case, %)	51 (7.53)	8 (15.69)	χ*^2^* =-2.014	0.044
Chronic obstructive pulmonary disease (case, %)	8 (1.18)	7 (13.73)	χ*^2^* =-6.024	<0.001
Chronic kidney disease (case, %)	9 (1.33)	1 (1.96)	χ*^2^* =-0.359	0.720
Cardiovascular diseases (case, %)	9 (1.33)	5 (9.80)	χ*^2^* =-2.623	0.009
Cancer (case, %)	17 (2.51)	1 (1.96)	χ*^2^* =-0.254	0.796
Other (case, %)	48 (7.09)	4 (7.84)	χ*^2^* =-0.284	0.777

According to Spearman correlation analysis, only the number of comorbidity was correlated positively with disease severity (Table 5), though no specific comorbidity correlated with disease severity (Table 5). Moreover, the number of comorbidities, NAFLD, CHB, and COPD were all correlated positively with virus negative conversion time (Table 5), and the number of comorbidities, CKD, CVD and hypertension correlated positively with prognosis (Table 5).

**Table 5:** Spearman correlation analysis of disease severity, virus negative conversion time, prognosis, age and comorbidities (*n* = 718).

**Variable**	**Disease severity (1** **=** **non-severe, 2** **=** **severe)**		**Virus negative conversion time (days)**		**Prognosis (1** **=** **survival,2** **=** **death)**	
	***r***	***P***	***r***	***P***	***r***	***P***
Age (year)	0.158	0.000	0.189	0.000	0.105	0.018
Number of comorbidities (0, 1, 2, 3 and more)	0.238	<0.001	0.225	<0.001	0.140	<0.001
Non-alcoholic fatty liver disease (1 = without,2 = with)			0.114	0.002		
Chronic hepatitis B (1 = without,2 = with)			0.089	0.017		
Chronic obstructive pulmonary disease (1 = without, 2 = with)			0.077	0.039		
Chronic kidney disease (1 = without,2 = with)					0.101	0.007
Cardiovascular diseases (1 = without, 2 = with)					0.081	0.030
Hypertension (1 = without, 2 = with)					0.087	0.020

While when the age was controlled, according to partial correlation analysis, the number of comorbidities was also correlated positively with disease severity (Table 6), the number of comorbidities and NAFLD were also correlated positively with virus negative conversion time (Table 6), and CKD, CVD and hypertension correlated positively with prognosis (Table 6). But there were also no longer any correlation between CHB, COPD and virus negative conversion time, and between the number of comorbidities and prognosis (Table 6).

**Table 6:** Partial correlation analysis of disease severity, virus negative conversion time, prognosis and comorbidities (when controlling for age) (*n* = 718).

**Control variable**	**Variable**	**Disease severity (1** **=** **non-severe, 2** **=** **severe)**		**Virus negative conversion time (days)**		**Prognosis (1** **=** **survival, 2** **=** **death)**	
		***r***	***P***	***r***	***P***	***r***	***P***
Age	Number of comorbidities (0, 1, 2, 3 and more)	0.147	<0.001	0.151	<0.001		
	Non-alcoholic fatty liver disease (1 = without, 2 = with)			0.106	0.005		
	Chronic kidney disease (1 = without, 2 = with)					0.117	0.002
	Cardiovascular diseases (1 = without, 2 = with)					0.088	0.018
	Hypertension (1 = without, 2 = with)					0.077	0.039

According to multiple stepwise regression analysis for disease severity, risk factors included age, the number of comorbidities and hyperlipidaemia (Table 7). Risk factors for virus negative conversion time were the number of comorbidities, hyperlipidaemia, NAFLD and COPD (Table 7). Furthermore, risk factors for prognosis were the number of comorbidities and CKD (Table 7).

**Table 7:** Multiple stepwise regression analysis of influencing factors for disease severity, clinical classification, coronavirus negative conversion time and prognosis (*n* = 718).

**Independent variable**		**B**	**Std. error**	**Beta**	***t***	***P***
Disease severity (1 = non-severe, 2 = severe)	Constant	0.989	0.012	-	84.1	<0.001
	Age	0.001	0.000	0.127	2.660	0.008
	Number of comorbidities (0, 1, 2, 3 and more)	0.048	0.007	0.254	7.027	<0.001
	Hyperlipidaemia (1 = without, 2 = with)	−0.043	0.021	−0.077	−2.118	0.035
Virus negative conversion time (day)	Constant	13.051	0.635	-	20.543	<0.001
	Number of comorbidities (0, 1, 2, 3 and more)	1.971	0.342	0.213	5.766	<0.001
	Hyperlipidaemia (1 = without, 2 = with)	−2.793	1.023	−0.102	−2.729	0.007
	Non-alcoholic fatty liver disease (1 = without, 2 = with)	2.121	0.806	0.099	2.631	0.009
	Chronic obstructive pulmonary disease disease (1 = without, 2 = with)	5.566	2.691	0.075	2.068	0.039
Prognosis (1 = survival, 2 = death)	Constant	0.992	0.025	-	183.925	<0.001
	Number of comorbidities (0, 1, 2, 3 and more)	0.013	0.003	0.148	4.024	<0.001
	Chronic kidney disease (1 = without, 2 = with)	0.088	0.031	0.105	2.864	0.004

### The Prediction of Number of Comorbidities on Disease Progression and the Outcomes of COVID-19 Patients

**Figure 8:**
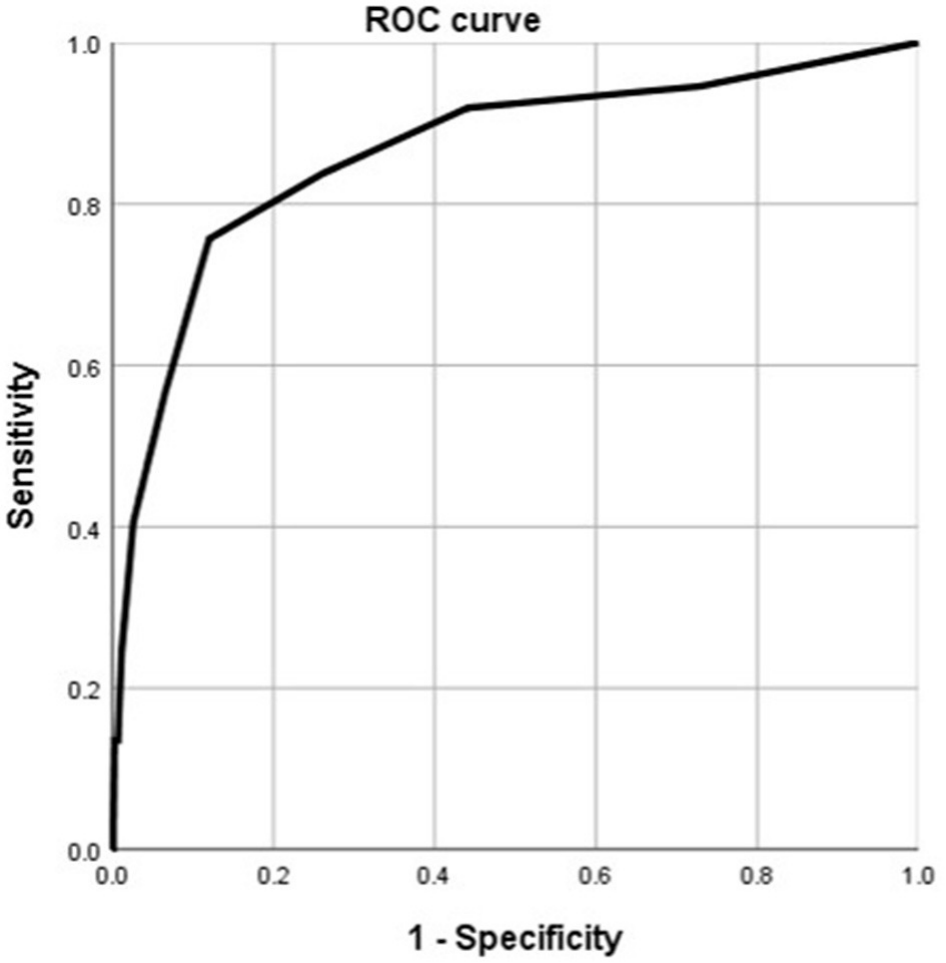
Using number of comorbidities for discriminating the severe cases from the non-severe patients (*n* = 718; non-severe and severe groups, *n* = 681 and 37, respectively). ROC analysis showing the performance of number of comorbidities in distinguishing severe cases from non-severe patients. ROC, receiver operating characteristic curve; AUC, area under the curve.

**Figure 9:**
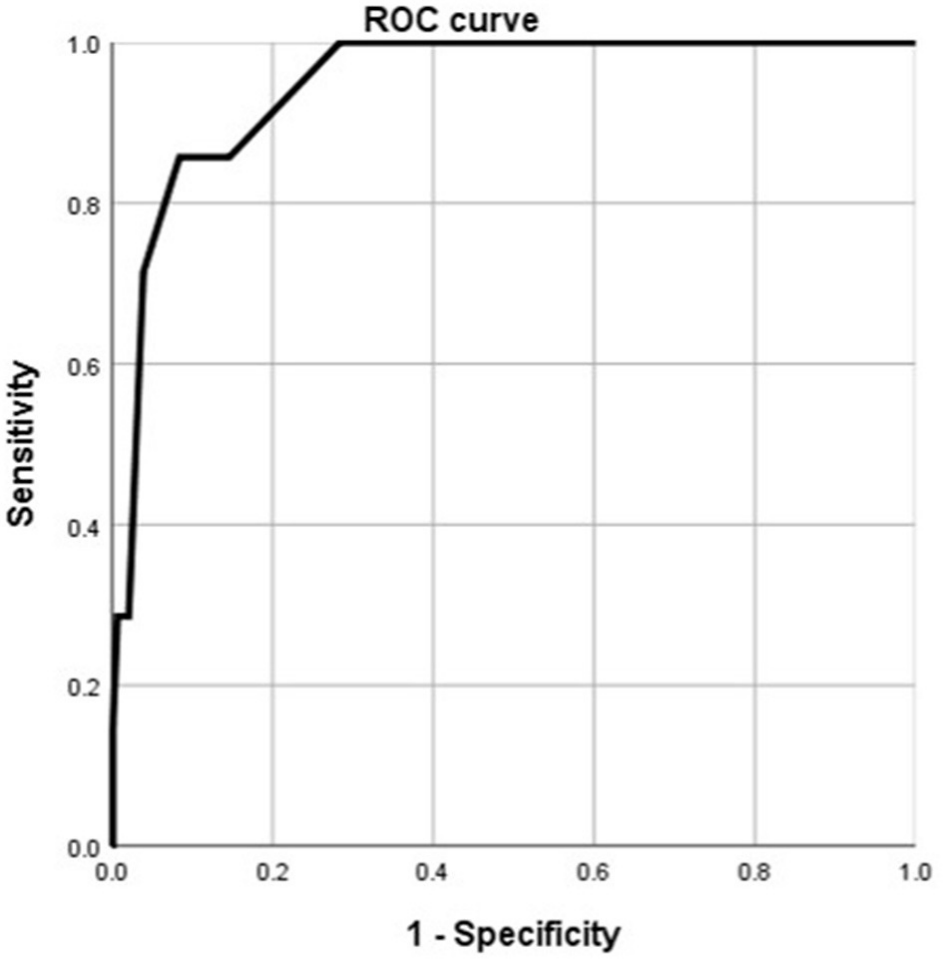
Using number of comorbidities for discriminating the dead cases from the surviving patients (*n* = 718; survival and dead groups, *n* = 710 and 8, respectively). ROC analysis showing the performance of number of comorbidities in distinguishing the dead cases from the surviving patients. ROC, receiver operating characteristic curve; AUC, area under the curve.

According to the ROC analysis, number of comorbidities could predict disease progression and patient outcomes (Tables 8, 9). The best cutoff point for distinguishing the severe cases from the non-severe cases was more than three comorbidities (Table 8). Its area under the curve was 0.864, (Table 8, Figure 8). Its sensitivity was 75.70% (Table 8). Its specificity was 88.00% (Table 8). The best cutoff point for distinguishing the dead cases from the survival cases was more than four comorbidities (Table 9). Its area under the curve was 0.947, (Table 9, Figure 9). Its sensitivity was 85.70% (Table 9). Its specificity was 91.60% (Table 9).

**Table 8:** The performance of various methods for distinguishing between the severe cases and the non-severe patients(*n* = 718).

**Variables**	**Cutoff point**	**AUC (95%CI)**	**Sensitivity**	**Specificity**	**False positive**	**False negative**
Number of comorbidities	3.5	0.864 (0.793~0.935)	75.70%	88.00%	24.30%	12.00%

*AUC, area under the curve; CI, confidence interval*.

**Table 9:** The performance of various methods for distinguishing between the dead cases and the survived patients(*n* = 718).

**Variables**	**Cutoff point**	**AUC (95%CI)**	**Sensitivity**	**Specificity**	**False positive**	**False negative**
Number of comorbidities	4.5	0.947 (0.893~1.000)	85.70%	91.60%	14.30%	8.40%

*AUC, area under the curve; CI, confidence interval*.

**Figure 10:**
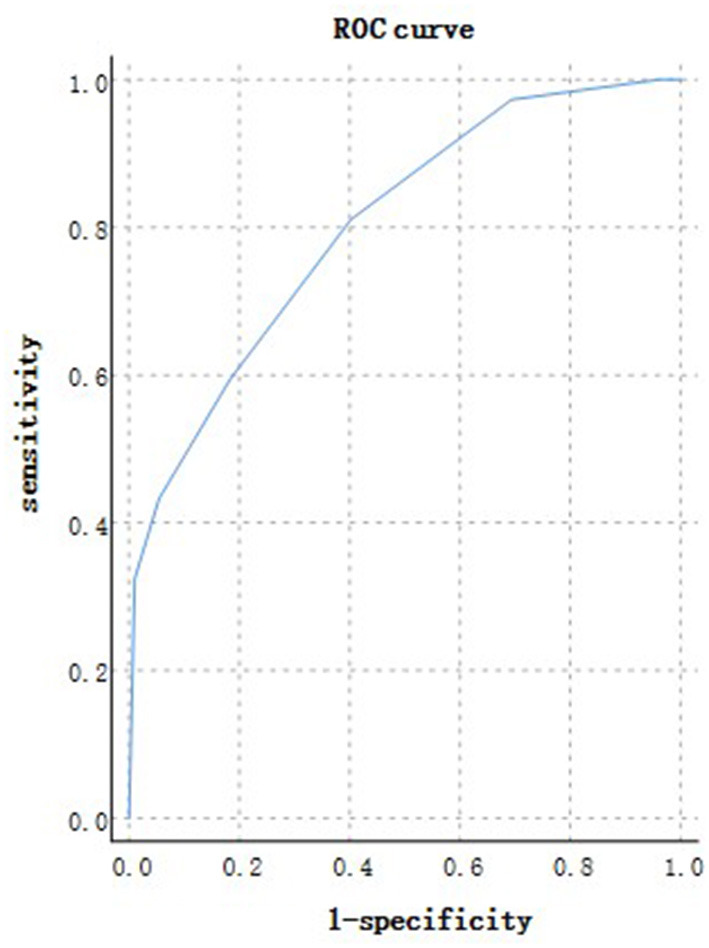
Using age for discriminating the severe cases from the non-severe patients (*n* = 718; non-severe and severe groups, *n* = 681 and 37, respectively). ROC analysis showing the performance of age in distinguishing severe cases from non-severe patients. ROC, receiver operating characteristic curve; AUC, area under the curve.

**Figure 11:**
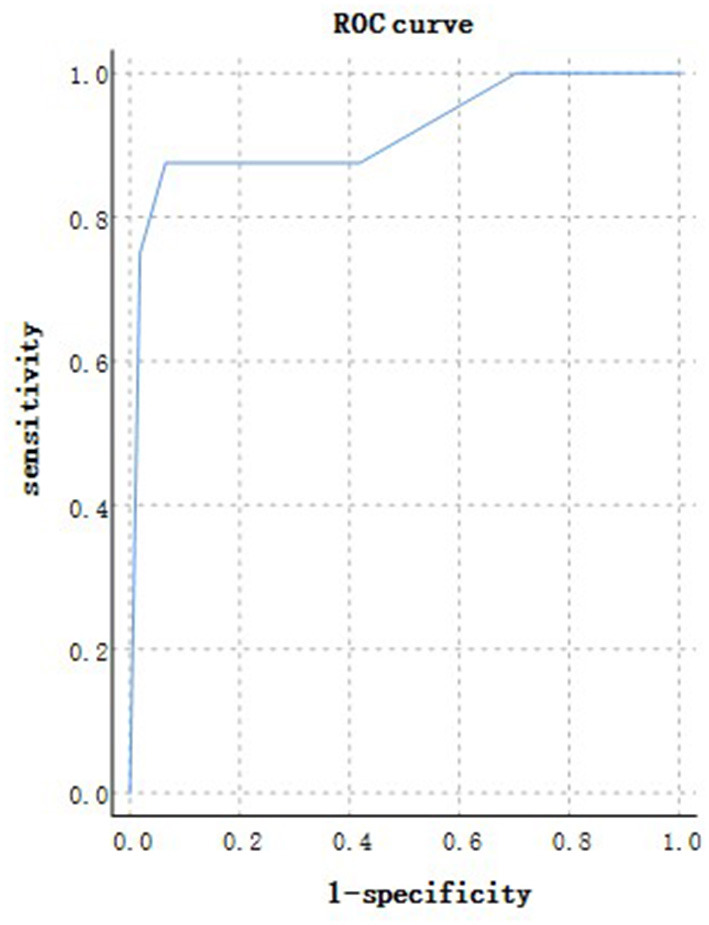
Using age for discriminating the dead cases from the surviving patients (*n* = 718; survival and dead groups, *n* = 710 and 8, respectively). ROC analysis showing the performance of age in distinguishing the dead cases from the surviving patients. ROC, receiver operating characteristic curve; AUC, area under the curve.

According to the ROC analysis, age could predict disease progression and patient outcomes (Tables 10, 11). The best cutoff point for distinguishing the severe cases from the non-severe cases was older than 45 years (Table 10). Its area under the curve was 0.801, (Table 10, Figure 10). Its sensitivity was 781.10% (Table 10). Its specificity was 59.80% (Table 10). The best cutoff point for distinguishing the dead cases from the survival cases was older than 65 years (Table 11). Its area under the curve was 0.918, (Table 11, Figure 11). Its sensitivity was 87.50% (Table 11). Its specificity was 93.50% (Table 11).

**Table 10:** The performance of various methods for distinguishing between the severe cases and the non-severe patients(*n*=718).

**Variables**	**Cutoff point**	**AUC (95%CI)**	**Sensitivity**	**Specificity**	**False positive**	**False negative**
Age (years)	45	0.801 (0.727~0.875)	81.10%	59.80%	18.90%	40.20%

*AUC, area under the curve; CI, confidence interval*.

**Table 11:** The performance of various methods for distinguishing between the dead cases and the survived patients(*n*=718).

**Variables**	**Cutoff point**	**AUC (95%CI)**	**Sensitivity**	**Specificity**	**False positive**	**False negative**
age (years)	65	0.918 (0.790~1.045)	87.50%	93.50%	12.50%	6.50%

*AUC, area under the curve; CI, confidence interval*.

## Discussion

In this COVID-19 cohort, the prevalence of severity was 5.16%, and mortality was 1.11%. Most patients with severe disease were older than 30 years, especially older than seventy, and most deaths occurred in those older than 70 years. Overall, age correlated with severity. We also found that age played a predictive role in distinguishing severe cases from non-severe patients and in distinguishing dead cases from surviving cases. Older than 45 and 65 years predict disease progression, a poor prognosis, respectively. This finding is consistent with the literature that old age is associated with the progression of COVID-19 and is an independent risk factor for progression (20) and that advanced age is a risk factor for a worse outcome in association with higher death rates (16–19).

Approximately 64.76% of the patients in this COVID-19 cohort had one or more comorbidities, and 37.88% had two or more. This is consistent with a report that one-third of patients have no comorbidity according to medical records, (14) but it is lower than the report that 70.7% of patients have one chronic condition and higher than the report that 20.9% patients have 2 or more (15). The reason may be that the characteristics of the study population were different. The median age of hospitalized patients was 63 years old (minimum: 18, maximum: 94) and the majority of cases were in the age range of 60~79 years old, all of patients were Iranian in literature (14). And 59.4% of patients aged between 20 and 39 years, 30.6% of patients aged between 40 and 59 years, only 7.4% of patients were older than 60 years, all of patients were Saudi Arabian in literature (15). While 53.76% of patients aged between 20 and 39 years, 34.96% of patients aged between 40 and 59 years, 8.77% of patients were older than 60 years, all of patients were Chinese in this cohort.

Further analysis found that severe cases had more comorbidities than non-severe cases; those who died had more comorbidities than surviving patients. An increased number of comorbidities correlated positively with disease severity and poor prognosis and was also an independent risk factor for progression and poor prognosis. This was consistent with previous findings that the number of comorbidities is a risk factor for a worse outcome (16–18, 21).

In this study, common comorbidities were mainly NAFLD, hyperlipidaemia, hypertension, DM, CHB, hyperuricaemia and gout; cancer, COPD, CVD, CKD and other comorbidities were not common. We found more types of comorbidities, especially metabolic diseases such as NAFLD, hyperlipidaemia, hyperuricaemia and gout in this COVID-19 cohort. The findings are not completely consistent with the report of common comorbidities in hospitalized patients of hypertension, CVD, DM, asthma, COPD, and other underlying diseases, (14) or the systematic review and meta-analysis of 76,993 patients that hypertension, CVD, DM, smoking, COPD, malignancy, and CKD, were most prevalent among patients with COVID-19 (13). But the findings are consistent with recent studies reported that 30.7–37.6% of COVID-19 patients from China had NAFLD, about 4.7 times higher than that of no-COVID-19 patients (22–24). Those NAFLD diagnosed of NASH increased 4.93 times the risk of COVID-19 (25).

Moreover, hypertension, DM, COPD, CKD and CVD were mainly present in patients with severe disease who were older than 50 years, especially among those 70 years old. Hypertension, CKD and CVD were common in patients who died and were older than 70 years. The number of comorbidities and age were correlated positively with disease severity, the number of comorbidities and NAFLD were correlated positively with virus negative conversion time, hypertension, CKD and CVD were primarily associated with those who died, and the above-mentioned correlation existed when age was controlled, and multiple stepwise regression analysis showed that the number of comorbidities and specific comorbidities and age were the risk factors for the disease severity and prognosis. These findings were not completely consistent with the literature report that DM and HBP or CVD are common underlying diseases related to death in hospitalized cases, (14) that COPD increases the risks of death and negative outcomes in patients with severe COVID-19, (26) that impaired renal function is an independent predictor of in-hospital death, (27) and that risk of death is associated with pre-existing hypertension, diabetes, or chronic kidney disease (21).

In this study we found that hyperlipidaemia was risk factor for the disease severity, and NAFLD and hyperlipidaemia were the risk factor for with virus negative conversion time. These findings are consistent with studies reported that the presence of NAFLD is independently associated with severe COVID-19 (24, 28–30) independent of obesity (31), especially those the presence of liver fibrosis (32) and with high serum interleukin-6 (IL-6) levels (33). NAFLD was an independent predictor of mortality or COVID-19 severity (34).

In hyperlipidaemia and NAFLD patients systemic overexpression of genes involved in SARS-CoV-2 entry and cleavage (such as FURIN, angiotensin I converting enzyme 2, and transmembrane serine protease 2), (35–37) pro-inflammatory M1 phenotype polarization of macrophages mediated by interferon, circulating levels of pro-inflammatory cytokines elevated, increased neutrophil-to-lymphocyte ratio with activation of the pro- interleukin-17 axis, and enhanced production of pro-coagulant molecules, (38–43) these pathways increase susceptibility of severe COVID-19 in NAFLD patients. Therefore chronic low-grade inflammation is suggested as the main leading process to trigger immune dysregulation, cytokine storm, and hypercoagulability in NAFLD patients with COVID-19 (43).

Even if the mortality rate of young people due to COVID, NAFLD and hyperlipidemia is low, young people (at least under 60 years old) often have NAFLD, hyperlipidemia and hyperuricemia, so some communication and advice should be given to them: it is not recommended to consume high-fat, high-glucose and fried foods, healthy and regular eating habits, healthy lifestyle, smoking cessation, alcohol restriction, regular exercise, etc.

In this study we found that number of comorbidities and age all played a predictive role in distinguishing severe cases from non-severe patients and in distinguishing dead cases from surviving cases. More than three and more than four comorbidities predict disease progression, a poor prognosis, respectively. Older than 45 and 65 years predict disease progression, a poor prognosis, respectively.

Based on these findings, not only three or more comorbidities, and some specific comorbidities, such as hypertension, CKD and CVD, but also age are related to progression and death in hospitalized COVID-19 patients.

Our study had several limitations. First, it was a retrospective, single-center study. Second, the number of severe cases, particularly deaths, was small. Despite these limitations, we report several novel findings: in addition to the common comorbidities reported in the literature, more types of comorbidities, especially metabolic diseases such as NAFLD, hyperlipidaemia and hyperuricaemia, were present in this COVID-19 cohort. Independently of age, two or more comorbidities, and some specific comorbidities, such as hypertension, CKD and CVD, are related to progression and death in hospitalized COVID-19 patients.

## Conclusion

In addition to the common comorbidities reported in the literature, there were more types of comorbidities, especially metabolic diseases such as NAFLD, hyperlipidaemia and hyperuricaemia, in this COVID-19 cohort. Not only two or more comorbidities, and some specific comorbidities, such as hypertension, CKD and CVD, but also age are related to progression and death in hospitalized COVID-19 patients. These findings provide a reference for clinicians to focus on the number and specific comorbidities, and age in COVID-19 patients to predict disease progression and prognosis.

## Data Availability Statement

The original contributions presented in the study are included in the article/supplementary material, further inquiries can be directed to the corresponding authors.

## Ethics Statement

The studies involving human participants were reviewed and approved by the Ethics Committee of the Public and Health Clinic Center of Chengdu approved this study (ethic approval number: PJ-K2020-26-01). Written informed consent was waived by the Ethics Commission of the designated hospital because this study is related to emerging infectious diseases. Written informed consent for participation was not required for this study in accordance with the national legislation and the institutional requirements. All participants understand that the information will be published without their child or ward's/their relative's name attached, but that full anonymity cannot be guaranteed. All participants understand that the text and any pictures or videos published in the article will be freely available on the internet and may be seen by the general public. The pictures, videos and text may also appear on other websites or in print, may be translated into other languages or used for commercial purposes. All participants have been offered the opportunity to read the manuscript.

## Author Contributions

DL, YZ, JK, DW, YM, GZ, HT, RZ, and LB: concept and design. DL, YZ, JK, DW, YM, and GZ: data acquisition, data analysis, and interpretation. YZ, JK, and DW: drafting the manuscript. YZ, JK, DW, HT, and LB: administrative, technical, or material support. HT, RZ, and LB: study supervision. All authors contributed to the article and approved the submitted version.

## Funding

This research was supported by the Thirteenth Five-Year Project on Tackling Key Problems of National Science and Technology (2017ZX10305501008), the Non-profit Central Research Institute Fund of the Chinese Academy of Medical Sciences (2020-PT330-005), the Sichuan Science and Technology Program (2020YFS0564), the Chengdu Municipal Science and Technology Bureau Science and Technology Huimin Major Demonstration Project (00092), the Sichuan Province Health Commission (17PJ070), the Chengdu Municipal Health Commission (2019079), and the Chengdu Science and Technology Bureau (2020-YF05-00191-SN).

## Conflict of Interest

The authors declare that the research was conducted in the absence of any commercial or financial relationships that could be construed as a potential conflict of interest.

## Publisher's Note

All claims expressed in this article are solely those of the authors and do not necessarily represent those of their affiliated organizations, or those of the publisher, the editors and the reviewers. Any product that may be evaluated in this article, or claim that may be made by its manufacturer, is not guaranteed or endorsed by the publisher.
